# State-trace analysis meets personality measurement: Why the Big Five tests are not based on five latent dimensions and how to fix them

**DOI:** 10.1371/journal.pone.0317144

**Published:** 2025-02-19

**Authors:** Johannes Titz

**Affiliations:** Institute of Psychology, Chemnitz University of Technology, Chemnitz, Germany; UCL: University College London, UNITED KINGDOM OF GREAT BRITAIN AND NORTHERN IRELAND

## Abstract

Unidimensionality is a fundamental yet often overlooked prerequisite for measurement. In the context of psychological measurement, the central question is whether a set of items can be logically reduced to a single latent factor. This study advocates for the application of state-trace analysis, an underutilized method from mathematical psychology, as a decisive tool to address this question. State-trace analysis provides a simple, general, and rigorous criterion for unidimensionality: monotonicity between item pairs. Identifying items within a factor that violate this criterion is straightforward, offering a practical approach to evaluating unidimensionality. This paper demonstrates the utility of state-trace analysis through exemplary applications within the framework of the five-factor model, analyzing data from the International Personality Item Pool-NEO-120 (*N* = 618, 000) and the NEO Personality Inventory–Revised (*N*_1_ = 857, *N*_2_ = 500). The findings reveal that maintaining the five-factor model requires significant revisions to numerous items, highlighting the potential of state-trace analysis to enhance personality measurement beyond existing methodologies. The paper concludes by discussing strategies to promote broader adoption of this method and how future designs in personality research can be tailored to effectively incorporate state-trace analysis.

## 1 Introduction

The purpose of this paper is to introduce a highly effective yet underutilized method for dimensionality analysis to personality measurement: state-trace analysis. Originally developed in the 1970s [1] and independently rediscovered in the 1980s [2], this method has only recently gained traction in mainstream psychology, largely due to improved software support. Despite its growing recognition, state-trace analysis remains relatively unknown in the domain of personality measurement, likely because its algorithms require adaptations to work effectively with non-experimental data. The potential of state-trace analysis is demonstrated through a reanalysis of the dimensional structure of two prominent Big Five questionnaires—the IPIP-NEO-120 [3] and the NEO-PI-R [4]. By presenting concrete examples where traditional methods fall short and highlighting cases that demand theoretical refinement or item modification, the aim is to showcase the method’s value and practical applicability.

State-trace analysis embodies the principle of “do one thing and do it well”. It is designed to address a singular but critical question with definitive precision: can two or more manifest variables (e.g., items) be reduced to a single latent variable? As will be discussed, no other method answers this question with such minimal and plausible assumptions. By leveraging its analytical rigor, state-trace analysis has the potential to transform how psychologists approach the design and evaluation of questionnaires, offering a robust framework for assessing unidimensionality in psychological measurement.

Before exploring the details of state-trace analysis, it is essential to address a broader question: Why is unidimensionality so important?

## 2 Unidimensionality

Unidimensionality is arguably the most fundamental prerequisite for measurement [5–8]. The concept can be intuitively illustrated through a simple visual analogy: if a construct is measurable, it can be represented on a single line, with objects (e.g., items or participants) positioned along it. Unidimensionality ensures that no additional lines are needed to capture the construct. This principle forms the basis of ordinal, interval, ratio, and absolute scales, making it a cornerstone of measurement theory. Furthermore, it aligns with the intuitive concept of the number line, often introduced to children as early as kindergarten.

When two constructs are involved, visual representation shifts to a two-dimensional coordinate system. For example, in physics, schoolchildren use two axes to represent the relationship between distance and time (e.g., to understand speed). Adding two distances measured in the same units is logical and meaningful, but combining distance and time is nonsensical because they represent distinct dimensions. Properly using two axes is essential to accurately capture and understand the phenomena at play.

The same principle applies to psychological measurement. Calculating a sum score (or factor score) for a construct like *intelligence* assumes that all items reflect the same underlying dimension. However, if the items actually measure distinct constructs—such as *concentration*, *attention*, or other related but separate abilities—the resulting sum score becomes ambiguous. In the best-case scenario, this adds random noise to the measurement, but in the worst case, it completely undermines construct validity.

The frequently observed low effect sizes in psychology (e.g., [9]) may partly stem from treating multidimensional constructs as if they were unidimensional. Even when a psychological hypothesis is valid, testing it becomes challenging if the assumed unidimensional constructs actually encompass multiple underlying dimensions. This misalignment not only obscures meaningful relationships but also diminishes the precision and reliability of psychological research.

Consider again the example of distance and time for traveling objects. While these variables are clearly distinct, they are often correlated: traveling a longer distance typically takes more time at a relatively constant speed. In Europe, distances between cities are commonly expressed in kilometers, whereas in the U.S., the author has observed that they are often described in terms of travel time (e.g., “It’s a three-hour drive”). If both variables are *z*-standardized to have the same mean and standard deviation, a factor analysis will misleadingly suggest a single factor with high loadings for both variables.

Using the factor score as a representation of “latent distance” introduces clear problems. When speed deviates from the norm—such as during a traffic jam, where time increases while distance remains constant—the measurements become highly unreliable. The concept of “latent distance” would lack consistency, varying each time based on travel conditions. Relying on such flawed measurements to test critical hypotheses is deeply problematic, as any failure to find significant results could easily be dismissed as a consequence of poor construct validity rather than a genuine flaw in the underlying theory (see [7] for a similar argument).

While this example is intentionally artificial and exaggerated, it highlights the critical importance of unidimensionality and the risks of treating multidimensional concepts as unidimensional. Without recognizing and accounting for distinct dimensions, measurements can become distorted, undermining their validity and interpretability.

The example also underscores the shortcomings of traditional methods of dimensional analysis. In particular, factor analysis proves insufficient because correlations capture linear associations rather than true unidimensionality. This limitation becomes clearer with the introduction of state-trace analysis, which offers a rigorous and precise definition of unidimensionality. Armed with this framework, it becomes apparent why alternative methods often fall short.

## 3 Applying state-trace analysis to personality measurement

The primary goal of state-trace analysis is to determine the minimum number of latent variables needed to explain observed empirical phenomena [10]. The method imposes theoretical constraints on the relationships between dependent and latent variables, thereby indirectly defining the conditions under which specific empirical patterns require a particular number of latent variables. State-trace analysis typically assumes monotonic relationships between a latent variable and two dependent variables [2]. A monotonic function consistently increases or decreases, although it may remain constant over certain intervals. Under these conditions, it follows that the relationship between the dependent variables must also exhibit monotonicity—a hypothesis that can be empirically tested. If the relationship between the dependent variables is found to be nonmonotonic, this indicates that two or more latent variables are required. Alternatively, it could mean that the initial assumption of monotonicity was incorrect. It is important to note, however, that this assumption is made *a priori*. While other types of relationships can be specified, monotonic relationships are often considered the most plausible for psychological data [11]. This point will be explored in greater detail later.

### 3.1 Formal argument and illustration

The formal argument, tailored to personality measurement (for a comprehensive treatment, see [2, 11]), unfolds as follows. Consider a latent variable *l* and responses to two questionnaire items, *i*_1_ and *i*_2_, both exclusively linked to this latent variable. It is reasonable to assume that the relationships between *l* and *i*_1_, as well as *l* and *i*_2_, exhibit monotonicity. Specifically, these associations should be monotonically increasing (isotonic): as the latent variable *l* increases, the dependent variables *i*_1_ and *i*_2_ should also increase, or at the very least, remain constant. In practical terms, this implies that, for instance, as latent extraversion increases, responses to an item such as “I like big parties” should never decrease on average. Formally,

$$\begin{array}{c} \frac{\;di_{1}}{\;dl}\geq 0\;\text{and}\;\frac{\;di_{2}}{\;dl}\geq 0 \end{array}$$ (1)

If this condition is met, the two items are also monotonically (or isotonically) associated. This relationship follows directly from the application of the chain rule:

$$\begin{array}{c} \frac{\;di_{2}}{\;di_{1}}=\frac{\;di_{2}}{\;dl}\frac{\;dl}{\;di_{1}}\geq 0 \end{array}$$ (2)

**Fig 1:**
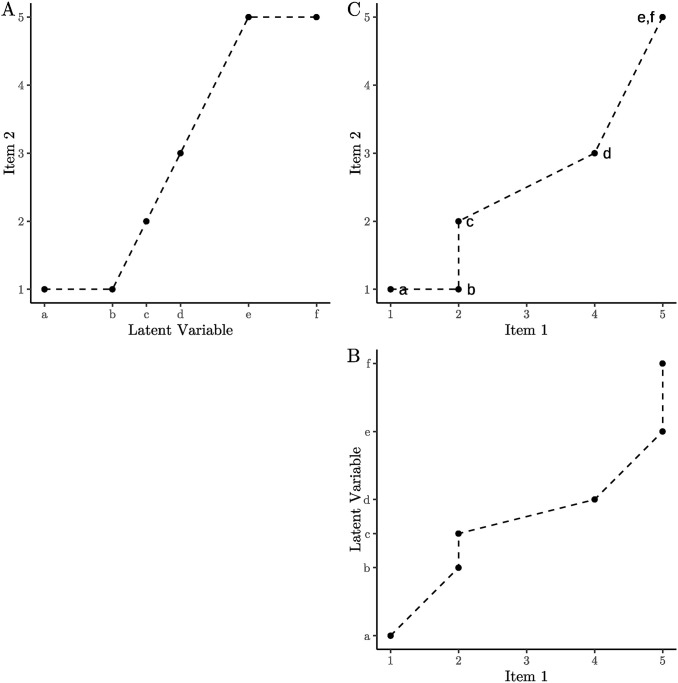
Illustration of a monotonic relationship between a latent variable and two questionnaire items. The relationships between the latent variable and the items are monotonic, which logically implies a monotonic relationship between the items themselves. This straightforward concept can be leveraged to analyze dimensionality. Dashed lines are included to aid in visualizing monotonicity.

For any value of *i*_1_, the differential change in *i*_2_ must be either zero or positive. Similarly, for any value of *i*_2_, the differential change in *i*_1_ must also be zero or positive. This relationship is visually illustrated in Fig 1.

In Fig 1B, the item is displayed on the *x*-axis, while the latent variable is plotted on the *y*-axis. This arrangement corresponds to the notation $\frac{\;dl}{\;di_{1}}$ and is particularly useful, as the values of Item 1 and Item 2 can be directly mapped onto Fig 1C, known as the *state-trace*. For instance, to locate Point *d* in the state-trace, extend lines from the values of Items 1 and 2 (shown in Fig 1A and 1B) corresponding to the latent value *d*. These lines should meet in Fig 1C, marking the position of Point *d*.

When items and latent variables exhibit an isotonic relationship, as shown in Fig 1A and 1B, the slope can only take one of two states: increasing (+) or remaining constant (=). Considering the interaction between two items (Item 1 and Item 2), four possible combinations of these states emerge: + =, = +, ++, and ==. For example, between Points *a* and *b*, Item 1 increases while Item 2 remains constant. From *b* to *c*, Item 2 increases while Item 1 remains constant. In the intervals between *c* and *d* and between *d* and *e*, both items increase, albeit at different rates. Finally, between *e* and *f*, both items remain constant. Throughout all these combinations, the relationship between the two items remains isotonic, as illustrated in Fig 1C.

It is worth noting that in Fig 1B, the slope between Points *b* and *c* is not defined because Δ*i*_1_ = 0. However, this issue is also reflected in the state-trace and poses no practical challenges. In typical nonexperimental psychometric studies, this scenario does not occur as mean values are typically analyzed, resulting in the averaging of points *b* and *c*.

In cases where deviations from monotonicity appear in the state-trace, the implication is the involvement of another latent variable. A unidimensional model becomes untenable. For example, if Point *b* were positioned one unit further to the right in the state-trace, at (3,1), an additional latent variable would be necessary to account for this pattern. Any attempt to modify Fig 1A and 1B to accommodate the new point (3,1), without violating monotonicity, proves unsuccessful.

To provide a more intuitive explanation, let us once again consider the analogy of travel distance and travel time. When speed remains relatively constant, the relationship between these two variables is monotonic: increasing travel time leads to an increase in travel distance. In such cases, the two variables can appear to be reducible to a single dimension. However, if speed varies, the relationship becomes non-monotonic. For example, moving point *b* to the coordinates (3, 1) implies that it took longer (3 units) to cover a shorter distance (1 unit) compared to point *c* at (2, 2). This discrepancy could be explained by an external factor, such as a traffic jam, which affects travel speed.

When a non-monotonic pattern emerges, it underscores the limitations of a unidimensional model, as such a model cannot fully capture or explain the observed data patterns. Instead, the researcher is compelled to identify and incorporate an additional factor to account for the complexity in the data. For instance, in the case of travel time and distance, it becomes evident that these variables cannot be reduced to a single dimension.

### 3.2 Monotonicity: One criterion to rule them all

Shifting point *b* to the location (3,1) introduces non-monotonicity and highlights a crucial distinction between monotonicity and correlation. Despite the shift, the correlation between the two items remains very high, decreasing only slightly from .96 to .89. However, the shift entirely undermines unidimensionality. This demonstrates a significant limitation of relying on correlations (or factor loadings) to detect dimensionality issues, as they fail to address the essential criterion of monotonicity. While exceptionally high correlations approaching 1 suggest monotonicity, such correlations are exceedingly rare in psychological data. In contrast, the more commonly observed low to moderate correlations offer little insight into whether monotonic patterns are present.

From the perspective of state-trace analysis, examining correlations to address dimensionality issues is justified only under a specific condition: when latent variables exhibit a linear relationship with manifest variables [11] (pp. 43–46). However, this assumption is both implausible and practically untestable due to the *problem of nomic measurement* [12] (p. 59).

This problem arises when attempting to measure a latent variable *l*, which is, by definition, not directly observable. To infer *l*, we rely on an observable manifest variable *m*, requiring knowledge of the functional relationship *l* = *f*(*m*). However, determining *f* empirically is impossible because it presupposes prior knowledge of *l*’s values—the very unknowns we aim to uncover. Consequently, assuming a linear relationship between *l* and *m* overlooks the fundamental problem of nomic measurement.

Similarly, item response theory (IRT) is limited as a method for assessing unidimensionality. In IRT, the probability of surpassing a specific scale point is modeled as a logistic function of the latent variable. However, this approach cannot be empirically validated because the values of the latent variable remain inherently unknown. The reliance on logistic functions is essentially a pragmatic solution to circumvent the problem of nomic measurement. While logistic functions offer mathematically convenient properties, there is no assurance that they accurately capture underlying psychological processes.

State-trace analysis provides a more elegant solution to the problem of nomic measurement. Rather than committing to a single function among countless possibilities, it defines a broad class of functions, typically monotonic ones (though other options are possible). This approach is advantageous because most psychologists would agree that the relationship between latent and manifest variables is generally monotonic. By adopting this inclusive framework, state-trace analysis avoids the restrictive assumptions inherent in linear or logistic functions, which represent only a narrow subset of plausible monotonic relationships.

But why should most psychologists agree that monotonic functions are appropriate? This is best explained with an example: Imagine a scenario where the relationship between latent extraversion and extraversion-related items is non-monotonic. In this case, there must be two points on the latent continuum where the following holds: individuals with lower latent extraversion (Point 1 on the continuum) would, on average, express greater agreement with a statement like *I talk to a lot of different people at parties* than individuals with higher extraversion (Point 2 on the continuum).

Such a formulation is conceptually perplexing and contradicts all established theories in personality psychology and psychometrics. A person with lower extraversion should, on average, never be more inclined to *talk to a lot of different people at parties* than someone with higher extraversion. Monotonicity is not merely a theoretical convenience; it is a reasonable assumption with significant face validity. In fact, it may be the only defensible assumption regarding psychological latent variables [11] (p. 30). Additionally, monotonic functions include linear and logistic functions, meaning methods like factor analysis and item response theory (IRT) naturally fall within this broader framework.

A common misconception among researchers new to state-trace analysis is the belief that psychology provides obvious counterexamples to monotonicity. A frequently given example, even by experts, is the Yerkes-Dodson law, which describes an inverted U-shaped relationship between arousal and performance. However, this example is fundamentally flawed because arousal and performance are clearly distinct variables. No psychologist would reasonably argue that arousal and performance constitute a single dimension; therefore, it is inappropriate to expect a monotonic relationship between them. The principle of monotonicity applies specifically within the context of unidimensionality, where a single latent variable governs the observed relationships.

Consequently, genuine counterexamples to monotonicity are rare, though they do exist. In certain complex models within cognitive psychology, monotonicity between latent and manifest variables does not hold [13]. The improper application of state-trace analysis in such cases can, and indeed has, led to erroneous conclusions [13]. However, these cases appear irrelevant in the context of questionnaire items, where non-monotonic relationships between latent and manifest variables are typically hard to imagine.

### 3.3 A personality measurement example

For a concrete illustration, let us consider a typical five-factor-model questionnaire. In the NEO-PI-R [4], each factor is represented by 48 questions, resulting in a total of 240 questions. It is postulated that the answers to each set of 48 questions corresponding to a factor are determined by a single latent variable. Consequently, each pair of questions (48 ⋅ 47 ⋅ 5 = 11, 280) is expected to exhibit a monotonic relationship. By testing this monotonicity condition, items that do not align with the corresponding dimension can be identified. Moreover, the method can be extended to test one item against several others simultaneously, such as comparing one item against a subscale or the entire factor.

### 3.4 Unidimensionality in hierarchical models

An essential consideration arises regarding how hierarchical models should be treated. Despite the NEO-PI-R being a five-factor model, it also incorporates a substructure, with each factor comprising six facets. It is plausible to treat each facet as a separate dimension or to focus solely on the overarching factors. Theoretically, even with a hierarchical substructure, all items belonging to one factor should exhibit a monotonic relationship.

Consider the following example: If extraversion increases, its facets should not decrease. An extraverted person, for instance, should not exhibit lower levels of *gregariousness* or *warmth* than an introverted person. Consequently, all items measuring the facets of extraversion—essentially all extraversion items—must also not decrease. In this sense, unidimensionality ignores hierarchical structures, focusing solely on whether objects (such as persons and items) can be arranged along a single continuum. If items from two facets (e.g., gregariousness and warmth within extraversion) cannot be aligned along such a continuum, it undermines the validity of treating these facets as part of a single dimension.

### 3.5 Experimental and nonexperimental approaches

The concept of state-trace analysis, while seemingly straightforward, has proven remarkably influential, with numerous applications (for a comprehensive overview, see [11]). However, to the best of my knowledge, it has not been adopted in personality research. One possible reason is that state-trace analysis, originally developed in cognitive psychology, is predominantly applied to experimental data. In such contexts, independent variables are manipulated to alter the values of a latent variable, and the relationship between dependent variables is then tested for monotonicity. The methodology and inferential framework of state-trace analysis were specifically designed for these experimental settings [11, 14].

Adapting state-trace analysis for use with nonexperimental data requires methodological adjustments and modifications to existing software tools. For cognitive psychologists primarily interested in causal arguments, these adaptations may offer limited appeal, as they do not align closely with their core research priorities.

In personality research, causality often takes a backseat to the primary focus: studying inherent differences between individuals. Attributes such as intelligence or extraversion are typically viewed as relatively stable within a person, making it impractical to significantly alter these traits in a short-term experiment and then restore them to their original state. Instead, the emphasis is placed on understanding variations between individuals.

As a result, the development of psychological questionnaires and tests in personality research is usually based on nonexperimental data. While state-trace analysis was originally designed for experimental contexts, its core principles remain relevant. Even without an experimentally manipulated independent variable, monotonicity between items can still be examined. When individuals naturally differ along a latent variable, there is no strict need to manipulate it. This approach is comparable to other analytical methods, such as regression, which can also be effectively applied to nonexperimental data, albeit with some differences in underlying assumptions and applications.

Undoubtedly, conducting experiments in personality research is valuable (as will be discussed later). However, it seems that deriving benefits from state-trace analysis is feasible by applying the method to already existing questionnaires, which are conventionally grounded in nonexperimental data. Thus, the primary focus is now directed towards testing questionnaire items for monotonicity in nonexperimental data. Although testing monotonicity may not seem inherently challenging at first glance, there are several methodological aspects that warrant careful consideration.

## 4 General method

When testing for monotonicity, it is crucial to consider the statistical nature of psychological behavior and experience. The objective is to determine whether monotonicity holds at the population level. Spurious violations of monotonicity are bound to occur with small samples due to sampling error. Pairwise tests for all items of one dimension demand a very large sample size because adjustment for *α* is necessary. Alternatively, testing one item against several others simultaneously is possible, but if violations of monotonicity are detected, identifying the specific item pairs causing the issue remains problematic. This is akin to an analysis of variance, where a significant result only indicates differences somewhere without specifying the particular groups involved.

To demonstrate the full potential of state-trace analysis in personality measurement, a sufficiently large dataset must be examined. A large sample eliminates any confusion that might arise from inference statistics, allowing for a clear focus on the effectiveness of the method itself. The International Personality Item Pool project [15] is an ideal test case for this purpose, offering extensive datasets on popular psychometric models. For example, in the IPIP project, a NEO-PI-R clone was developed and validated in a large study with over 600,000 participants [3].

However, exploring the performance of more established psychological questionnaires is equally intriguing. Traditionally, these questionnaires are proprietary, and large datasets for them are typically unavailable. In such cases, it is vital to have a statistical test of monotonicity, introduced in the next section, to ensure the reliability of the results.

### 4.1 Bootstrap test of monotonicity

[11] (p. 55) delineate a double-bootstrapping procedure for testing the monotonicity of two dependent variables. This methodology is rooted in the work of [16], who provided a more rigorous description of the statistical test and extensively studied it through simulations (see also [14]). The test, known for recovering latent structures effectively and exhibiting robust power, has become the standard procedure for conducting state-trace analysis [10]. An alternative approach was presented by [14], but its application is challenging as it currently lacks a significance test. A key advantage of [10]’s test lies in its flexibility, allowing application to various situations. The procedure is intricate and is most effectively understood through a study of either a programming implementation or the pseudo-code by [16] (p. 10). For completeness, a verbal description of the main steps is provided here.

It is important to recognize that the values of an item in psychological questionnaires are typically discrete, which simplifies the problem. However, this discreteness may render Step 4 in the following procedure somewhat problematic. To address this, a parametric bootstrap was performed for comparison, yielding results that were highly consistent with the original approach.

The procedure for testing a pair of items (*i*_1_ and *i*_2_) involves the following steps:

1. Fit the best monotonic model to the data and calculate the residual deviance, denoted as $\omega =\sum_{j=1}^{k}w_{j}{\left(y_{j}-y_{j}^{*}\right)}^{2}$. In this context *y* represents the observed average *i*_2_ value and y* represents the most likely monotonic average *i*_2_ value, for every value of *i*_1_ (ranging from *j* = 1 to *j* = *k*, for a Likert scale from 1 to 5). The term *w*_*j*_ corresponds to the weight.
2. With replacement, draw a bootstrap sample of *i*_2_ grouped by the discrete values of *i*_1_.
3. Fit a monotonic model to the bootstrapped data.
4. Shift the bootstrapped data to the monotonic model. To accomplish this, the *i*_2_ values are shifted by the difference between the average *i*_2_ value and the optimal *i*_2_ value, again grouped by the discrete *i*_1_ values.
5. Bootstrap from the shifted, now monotonic, data (second bootstrap sample).
6. Calculate the residual deviance for the bootstrapped-shifted data.
7. Repeat Steps 2 to 6 often (depending on the precision needed).
8. Calculate the relative frequency of the bootstrapped deviance values being larger or equal to the empirical deviance value. This is the *p* value, which can be compared with *α*.

Note that the procedure is non-parametric and there are no specific assumptions due to the bootstrap.

Executing this procedure with large datasets is exceptionally resource-demanding. Some of the computations would have taken weeks, even with our relatively modern machine boasting 32 threads (Dell R6515). To mitigate computing time, a step-wise approach was adopted, involving the generation of a small number of bootstrap samples for each pair. Subsequently, pairs with a small *p* value were identified and subjected to further testing with additional bootstrap samples to increase precision. The required number of samples is determined by the *α* value. Smaller *α* values necessitate a higher number of samples to achieve the desired precision for the *p* value.

When dealing with a relatively small dataset, testing all possible item pairs might not be practical. The accumulation of *α* errors and the subsequent need for correction can significantly diminish the statistical power. For example, in the case of 48 items belonging to one factor (as in the NEO-PI-R), the number of tests escalates to 48 ⋅ 47 = 2, 256 (for one factor alone). A more sensible approach may involve testing each item against the entire factor simultaneously, effectively reducing the number of tests to 48.

### 4.2 Aggregated bootstrap

If one wishes to test a single item against several others, an extension of the described bootstrap procedure can be employed. Continuing with the example of 48 items within one factor, testing one item against the factor using the described bootstrap procedure results in 47 residual deviance values. Each deviance value corresponds to a bootstrapped sampling distribution. The final test statistic is obtained by summing these 47 residual deviance values. The sampling distribution is constructed by aggregating the bootstrapped residual deviance values across each run of the bootstrap procedure (for a similar approach, see [11], p. 54).

### 4.3 Handling rarely used scale points

For small datasets, it is possible that certain scale points of items have very few values. For instance, if one of the answers is relatively extreme or socially undesirable, it might be chosen by only a few participants. In the subsequent analyses, any answer category with fewer than five participants was excluded before conducting the analysis. While a cutoff of five may seem relatively low, it is crucial to consider that there are typically numerous items per factor. For instance, the NEO-PI-R includes 48 items per factor. Even if a specific scale point for an item is endorsed by only five participants, this still provides 235 data points. If all these responses show significant deviations from monotonicity, it is essential to ensure such cases are not overlooked.

Having outlined the general methodology, the empirical section of the paper is presented in two parts: (1) the analysis of the IPIP-NEO-120 based on a large internet sample, and (2) the analysis of the NEO-PI-R based on the Eugene Springfield student sample and a Netherlands student sample.

Notably, this paper was not preregistered; however, the data and analysis scripts employed are openly accessible for reference and scrutiny.

## 5 Part I: IPIP-NEO-120

The IPIP-NEO-120, although less established than older instruments like the NEO-PI-R, serves as a suitable starting point for introducing state-trace analysis to personality research. This choice is supported by several key considerations.

Firstly, the extensive dataset from the IPIP-NEO-120 validation study (N = 618,000) serves as a robust foundation for showcasing the method’s advantages. Given the substantial sample size, the intricacies of inferential statistics become less pivotal, enabling a more straightforward visual assessment of monotonicity violations. The argument, in this context, leans heavily on pure logic, as monotonicity violations are discernible to the naked eye, emphasizing the inadequacy of a single dimension to explain the observed data patterns.

Secondly, the IPIP-NEO-120 is non-proprietary, enabling open discussion and modification of its items in publications without copyright constraints.

Lastly, designed as an alternative to the NEO-PI-R, the IPIP-NEO-120 shares structural similarities, making it likely that both questionnaires face similar issues [17] (p. 270).

The IPIP-NEO-120, introduced by [3], represents the latest addition to the International Personality Item Pool’s (IPIP) collection of questionnaires aligning with the five-factor model. It was developed as a condensed version (120 items) of the IPIP-NEO (300 items). Both instruments share a common objective—to provide a free alternative to the copyrighted NEO-PI-R. This alignment is evident in the IPIP-NEO-120’s structure, organized into 30 facets, with six facets corresponding to each of the five factors. The facet names closely mirror or are identical to those of the NEO-PI-R. The items in the IPIP-NEO-120 closely mirror those in the NEO-PI-R, with some items being nearly identical.

The IPIP-NEO-120 was developed and validated with a high degree of transparency. [3] detailed the item-selection process, drawing from the IPIP-NEO (300-item version) with a sample of approximately 20,000 participants. Subsequent validity assessments were conducted across four samples, including two sizable internet samples with about 307,000 and 618,000 participants, respectively. The focus of the current analysis is on the larger sample, which exclusively evaluated the IPIP-NEO-120 (the smaller one measured several other constructs). In terms of reliability, the facets demonstrated acceptable values (*α* above .6), particularly noteworthy given that facets were measured with only four items. Correlations between IPIP-NEO-120 facets and NEO-PI-R facets averaged .66 (.91 when corrected for attenuation due to unreliability). The congruence between NEO-PI-R factors and IPIP-NEO-120 factors, expressed by Tucker’s coefficient, was .93, .97, .92, .97, and .95 for neuroticism, extraversion, openness, agreeableness, and conscientiousness, respectively (denoted N, E, O, A, and C in item numbers). These high values indicate a strong alignment, supporting the conclusion that the IPIP-NEO-120 serves as a nonproprietary counterpart to the NEO-PI-R.

The only issue identified was that some facets exhibited loadings on multiple factors. However, this is not unique to the IPIP-NEO-120 and is a phenomenon observed in other questionnaires, including the NEO-PI-R (see exemplary factor loadings in [18–21]). It is not a primary concern in the context of this paper, as the focus of state-trace analysis is on assessing the unidimensionality of individual factors. While alternative applications of state-trace analysis are conceivable, commencing with the simplest case is a logical starting point.

Further validation of the IPIP-NEO-120 was conducted recently by [17], yielding similar results. In their study, the confirmatory factor analysis supported the five-factor structure and facet substructure. Although the facet modesty exhibited a relatively low loading on the agreeableness factor (0.14), and openness displayed a somewhat loosely structured facet, the authors concluded that the IPIP-NEO-120 “is sufficiently structurally robust for future use” (p. 260). To scrutinize the foundation of this claim, the unidimensionality of each factor of the IPIP-NEO-120 will be analyzed in the following sections.

### 5.1 Method

The IPIP-NEO-120 comprises 120 items, with 24 items allocated to each dimension and four items for each of the six facets within a dimension. This leads to a total of 2,760 tests, resulting in an adjusted *α* of approximately $\frac{0.05}{24\cdot 23\cdot 5}=1.81e-5$. To obtain a sufficiently precise estimate of the *p*-value, at least 55,200 simulation runs are required for each tested pair (100,000 were conducted in the final step).

The original data set can be obtained from the Open Science Foundation website in connection with the corresponding publication by [3]. The dataset has undergone standard cleaning procedures (for details see [3], p. 83), which involved the exclusion of duplicates, responses indicative of inattentiveness, and instances with excessive missing values. Given the substantial size of the dataset, the power of all tests is anticipated to be very high, and there are no other technical concerns. It is worth noting that the dataset lacks a well-defined population, which is not a significant concern at this stage since the primary focus is on demonstrating the capabilities of state-trace analysis. Furthermore, the dataset has been employed in validation studies, affirming the functionality of the questionnaire and its adherence to a five-dimensional structure in the sample.

Due to the dataset’s size, even relatively weak violations can attain statistical significance. Consequently, it is pertinent to assess the strength of these violations. A network graph serves as an ideal tool to visualize this information, where items are depicted as nodes, and statistically significant violations are represented as edges between these nodes. The width of an edge reflects the strength of a violation (square root of deviance), facilitating the identification of the most problematic items in the questionnaire concerning unidimensionality.

### 5.2 Results

**Fig 2:**
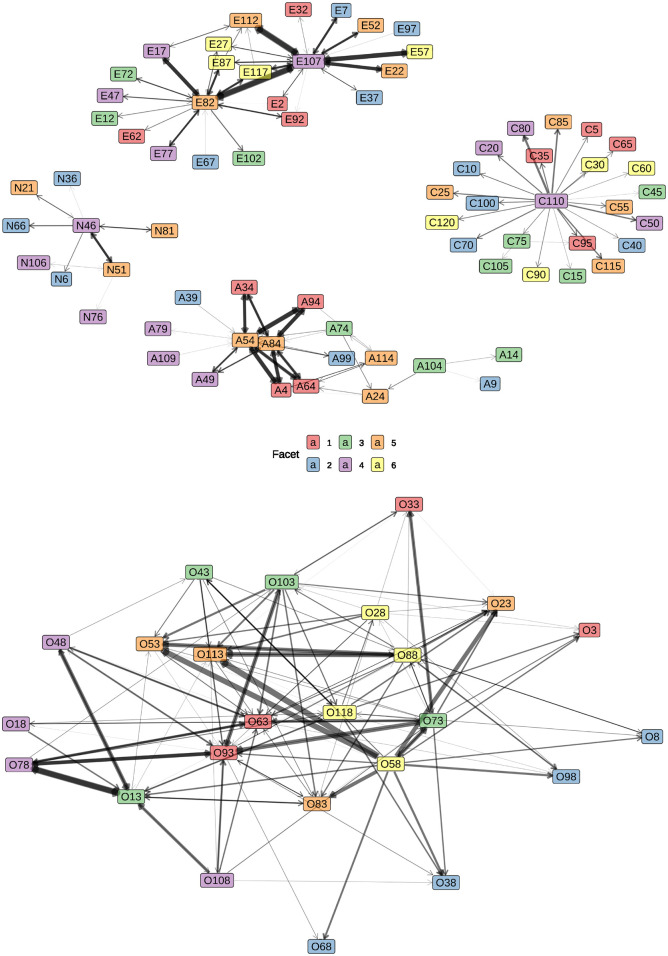
International personality item Pool-NEO-120 network graph of monotonicity violations for all five factors. Item designations A, C, E, N, and O represent the factors agreeableness, conscientiousness, extraversion, neuroticism, and openness, respectively. The six facets of each factor are differentiated by colors, and the width of the arrows in the network graph represents the strength of monotonicity violation (indicated by the square root of the deviance).

Overall, 267 statistically significant violations of monotonicity were identified. The network graph of violations is presented in Fig 2. Arrows indicate the direction of violation. For example, on the top left (neuroticism items), N66 is violated by N46, but not the other way around, whereas N51 and N46 are violated in both directions. It is important to note that in a nonexperimental design, a violation can occur in only one direction. When averaging, the relationship between *x* and *y* is not the same as between *y* and *x*.

Discussing all violations in a single paper is impractical, and instead, some of the most severe violations will be exemplarily discussed in detail to understand the underlying semantic problems and the advantages of state-trace analysis. Studying the number and width of the arrows in the network graph reveals the most problematic items: A54 and A84 for agreeableness, C110 for conscientiousness, E82 and E107 for extraversion, and N46 for neuroticism. For openness, many items present issues, but facets 5 (intellect) and 6 (liberalism) stand out.

#### 5.2.1 Confusing modesty with low self-confidence

**Fig 3:**
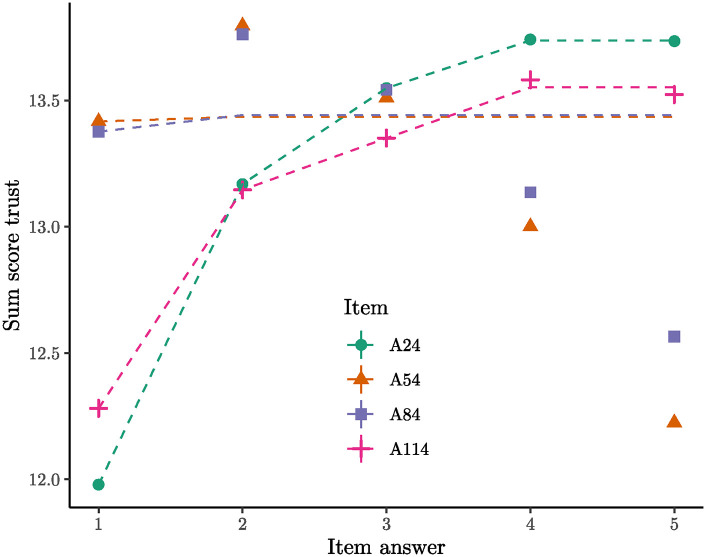
Trust sum score plotted against all modesty items. The 95% confidence intervals were plotted but they are not visible because they are smaller than the data points. The dashed lines represent the best fitting isotonic models as a visual guide. For Items A54 and A84 the empirical data is completely off the isotonic model.

Items A54 (“I think highly of myself”) and A84 (“I have a high opinion of myself”) are nearly identical and belong to the modesty facet. Both items exhibit a clear pattern in the network graph, as they are entirely incompatible with Facet 1, trust. To comprehend this, the sum score for trust was calculated and plotted against all modesty items. As shown in Fig 3, the relationship for Items A54/A84 with trust is non-monotonic. From answer options 2 to 5, the relationship decreases significantly, indicating a severe violation of unidimensionality.

Note that the 95% confidence intervals are so small that they are not visible. Constructing useful confidence intervals is challenging due to the extremely small standard errors, making them practically invisible on the scale. The implication is that the observed data pattern is highly unlikely to occur by chance in the context of the population from which the sample was drawn.

Participants who think highly of themselves (low values on A54/A84) tend to trust other people. In other words, individuals with high self-esteem are not very modest but exhibit a tendency to trust others. This contradicts the Big Five model’s claim that modesty and trust are positively related; high agreeableness should result in more trust and more modesty, not more trust but less modesty.

For the other two modesty items (A24: “I believe that I am better than others” and A114: “I boast about my virtues”), the relationship with trust is largely monotonic. It appears that items A54 and A84 are not measuring the same aspect of modesty as items A24 and A114. Persons can have a high opinion of themselves (A54 and A84), but this does not necessarily mean that they think they are better than others (A24) or brag about being better (A114). Although all four items sound similar, they seem to measure two distinct things.

**Fig 4:**
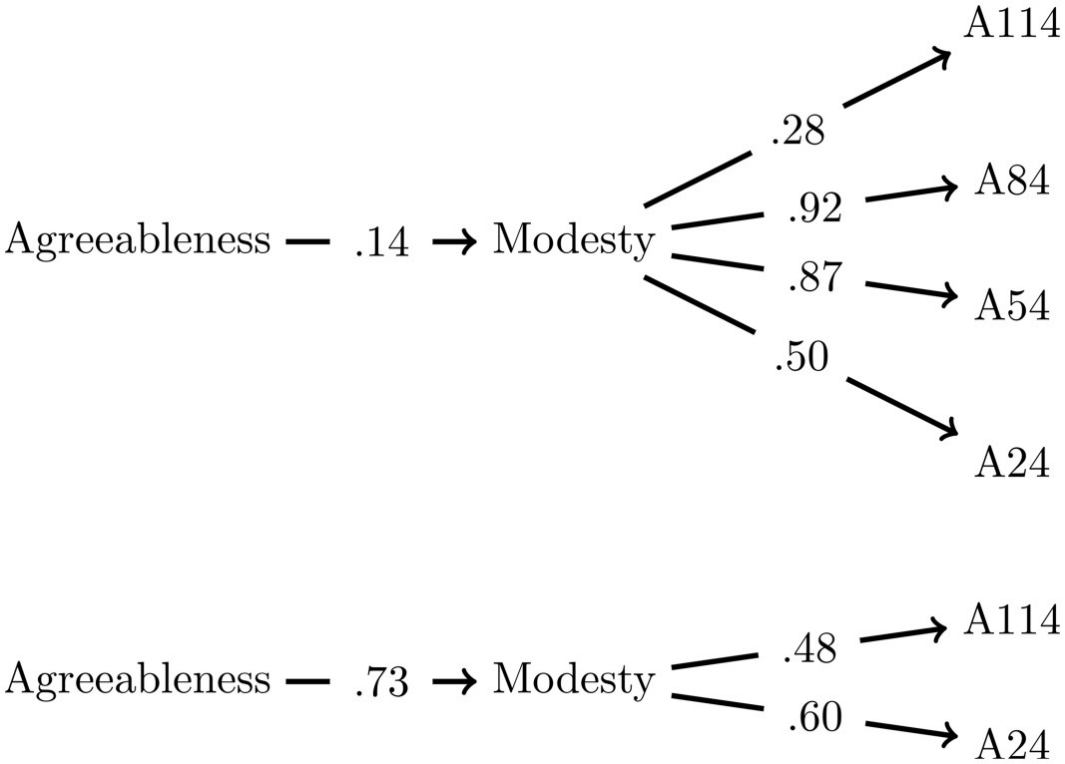
Two versions of the facet modesty in the Big Five structural equation model. In the upper model, items A54 and A84 are included, while in the lower one, they are excluded. The factor loading of modesty changes from 0.14 to 0.73. The full models (with all facets) for both cases are shown in S1 and S2 Figs respectively.

Items A54 and A84 exhibit such severe violations of monotonicity that there should also be problems in factor analysis. A structural equation model for the factor agreeableness was computed and is presented in S1 Fig. The results closely mirror those of the original authors [17], who analyzed a subset of the same data set, focusing on the country USA. The modesty segment of the model is depicted in Fig 4 (top). The factor loading for modesty on the factor agreeableness is a mere .14. However, the underlying problem is not immediately apparent. The factor loadings do not directly disclose that A54 and A84 are incompatible with trust. The original authors [17] did not reach the conclusion that these two items must be altered or removed. In fact, it may seem counterintuitive to many to discard the best loading items (A54 loads .87 and A84 loads .92), and they may choose to eliminate item A114 (with a factor loading of .28) instead. However, state-trace analysis streamlines the interpretation: A54 and A84 load heavily on a facet that is not functioning well.

After the removal of Items A54 and A84, the factor loading of modesty increases from 0.14 to 0.73, contributing an additional 8% to the explained variance in agreeableness. This represents a substantial improvement, and it is surprising that this straightforward solution has not been suggested earlier. The probable reason for this oversight is that factor analysis does not inherently demand the removal of Items A54 and A84. [17] speculated about the incompatibility of modesty with agreeableness as a whole, suggesting that the modesty items might contain a higher degree of social desirability, consequently reducing the variance of the facet. Additionally, they proposed that modesty could be associated with the proposed sixth factor (honesty-humility) in the extended Big Five model HEXACO [22]. The resolution provided by state-trace analysis is much simpler: If you aim to measure modesty, avoid confusing it with low self-confidence.

Although this result is impressive, the use of state-trace analysis does not always improve the factor structure in factor analysis. This is because state-trace analysis does not take into account correlations or factor loadings. A weak factor loading does not necessarily indicate a violation of monotonicity, and a relatively high factor loading does not automatically indicate monotonicity. A notable example of the latter is item C110, which is examined next.

#### 5.2.2 Quick but not necessarily dirty

One of the most intriguing items from the network graph is C110, “I put little time and effort into my work”, belonging to the facet achievement-striving. This item stands out by being inconsistent with all the other 23 items of the conscientiousness factor, an occurrence unique in this dataset.

It is noteworthy that Item C110 loads with 0.65 on its facet, and the facet loads with 0.84 on the factor. These robust factor loadings render it exceedingly challenging to identify Item C110 as problematic within the traditional factor-analytical framework [3, 17]. However, state-trace analysis unequivocally rejects the unidimensional hypothesis. In its present form, the item cannot coexist as part of a unidimensional scale with the other 23 items.

**Fig 5:**
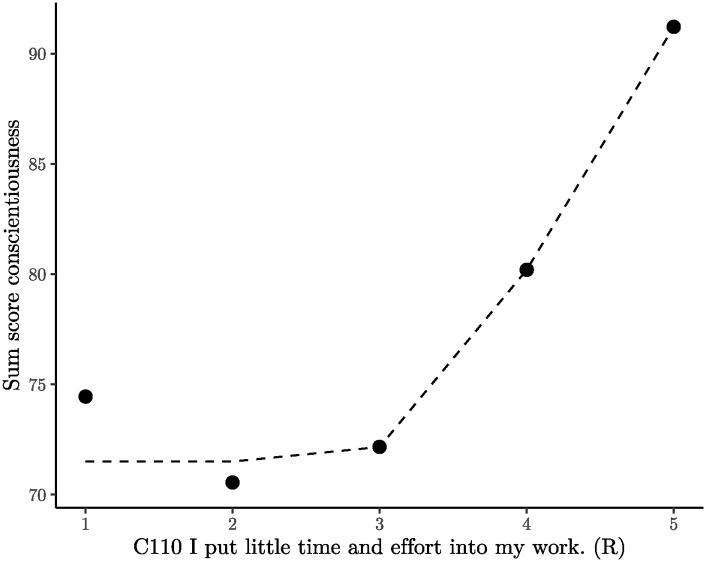
Conscientiousness sum score plotted against item C110. The 95% confidence intervals were plotted but they are not visible because they are smaller than the data points. The sum score excludes Item C110. The dashed line represents the best fitting isotonic model as a visual guide.

The underlying issue becomes evident when examining Fig 5, where Item C110 is plotted on the *x*-axis, and the sum score of the remaining 23 items of the factor is on the *y*-axis. Participants who strongly agree (recoded scale point 1) with the statement “I put little time and effort into my work” exhibit higher conscientiousness than those who merely agree or express neutrality.

The behavior of this item can be compared to a scale that effectively measures weight (sum score) within the range of 70kg to 100kg. However, when the weight falls below 70 kg, the scale becomes defective and consistently displays values around 75 kg, indicating a systematic bias. Despite its high factor loading, the item is in dire need of revision, either by repair or replacement.

Until the item is rectified, a potential solution is to code answer option 1 as a missing value. This adjustment increases all correlations between Item C110 and the 23 other items, though not significantly. The factor loading of C110 on the facet achievement only increases marginally from 0.65 to 0.71. However, eliminating a scale point is not equivalent to fixing an item. If the item were functioning correctly, the sum score for scale point 1 in Fig 5 should be below or around 70. This change should boost the factor loading more effectively than merely excluding scale point 1.

How could Item C110 be corrected? Since it contains two statements (“I put little time into my work” and “I put little effort into my work”), each may lead to different interpretations. One suggestion is to separate the two statements and investigate which one is problematic. Hypothetically, individuals who put minimal time into their work can still perform adequately if they are highly competent. Such individuals might not consider themselves to be very conscientious, but they would probably not consider themselves to be below average conscientious either.

The examination of Item C110 highlights that applying state-trace analysis to questionnaires is a thoughtful and nuanced process. Unlike the previous section, where the violations of Items A54 and A84 were so severe that removal was the only viable option, Item C110 generally behaves well, and outright removal would be imprudent. Delving into the semantic distinctions between “little effort” and “little time” in a dedicated study could enhance the properties of Item C110 at reasonable cost. Such an investigation would also contribute to a more nuanced psychological understanding of the facet achievement-striving.

Traditional factor analysis based on Pearson correlations is fundamentally limited in identifying the problematic nature of Item C110 because factor loadings can be high despite non-monotonicity. A comparable situation arises with non-monotonicity for moderately sized factor loadings. The exploration of such scenarios, particularly when the relationship between two variables is U-shaped, will be addressed in the next section.

#### 5.2.3 Can conservatives be intellectual?

**Fig 6:**
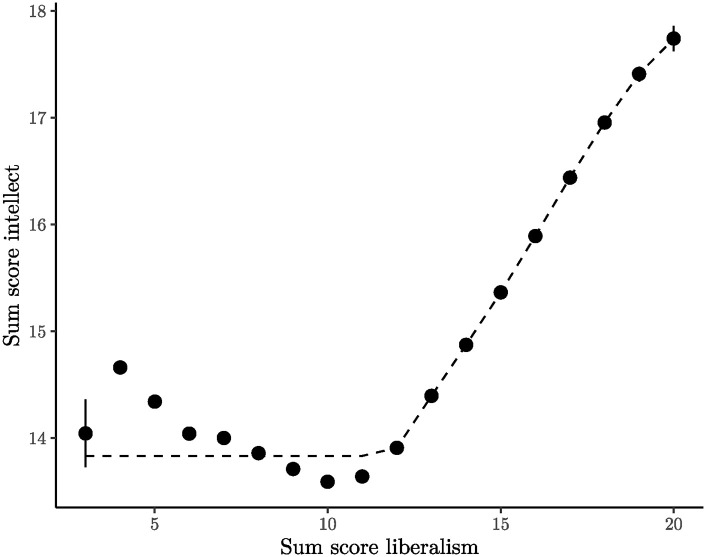
Intellect sum score plotted against liberalism sum score. Liberalism sum scores of 1 (with 2 participants) and 2 (with 31 participants) were excluded due to their limited sample size. Although 95% confidence intervals were drawn, they remain unnoticeable (except for the liberalism sum scores of 3 and 20) because they are narrower than the plotted data points. The dashed line represents the best-fitting isotonic model as a visual guide.

The network graph exposes that openness to experience emerges as the most challenging factor. Multiple instances of unidimensionality violations are evident, with the most frequent and severe transgressions occurring between the liberalism facet (O28, O58, O88, O118) and the intellect facet (O23, O53, O83, O113). In an effort to discern the specific issue, the cumulative scores for both facets were computed. The relationship between these facets demonstrates a non-monotonic trend (see Fig 6). Participants positioned in the middle (scoring 10 points, indicative of a neutral stance between conservatism and liberalism) exhibit the lowest intellect scores. A sharp increase is observed when moving to the liberal side, but somewhat surprisingly, there is also a slight increase when moving to the conservative side, thereby violating monotonicity.

To date, psychologists using factor analytic methods seem to have overlooked this issue because of the divergence between monotonicity and correlation. Despite the non-monotonic nature of the relationship between the two facets, a positive correlation of .18 persists. While not exceptionally high, this value is still sufficient to produce reasonable results in factor analysis. Specifically, liberalism has a factor loading of .36 on openness to experience, and intellect has a loading of .71. These results suggest that there is no obvious reason to exclude the facet of liberalism based solely on the results of factor analysis.

In contrast, state-trace analysis reveals a critical revelation: liberalism and intellect cannot form a unified dimension within this dataset. This discrepancy poses a significant conceptual challenge that needs to be addressed. The central question is whether the attributes of liberalism and intellect can ever form a monotonically related relationship. A compelling counterexample is provided by prominent conservative figures in politics who actively engage in both oral and written intellectual debate. Similarly, priests, who are by definition conservative, also exemplify intellectual pursuits.

Thus far, the presented examples illustrate how state-trace analysis can offer novel insights that would be challenging or even impossible to attain through factor analysis alone. The last example demonstrates how state-trace analysis can provide valuable assistance with items that pose ambiguity in the context of factor analysis.

#### 5.2.4 “Taking it easy” is a sign of extraversion and introversion

**Fig 7:**
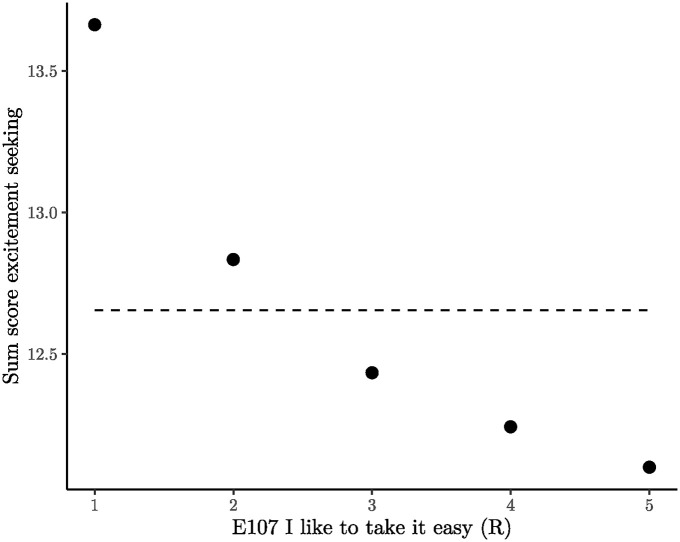
Excitement seeking plotted against item E107. The 95% confidence intervals were drawn but they are not visible because they are smaller than the data points. The dashed line represents the best fitting isotonic model as a visual guide.

Item E107 (“I like to take it easy”) is inconsistent with 14 other extraversion items and is completely incongruent with Facet 5, excitement Seeking. As shown in Fig 7, the relationship between this facet and E107 is negative. According to the five-factor model, participants who express a preference for “taking it easy” are expected to be less extraverted; however, they paradoxically exhibit higher scores on excitement seeking, indicating greater extraversion. This contradiction implies an inherent conflict: One cannot be less extraverted and more extraverted at the same time.

The semantics of item E107 are nuanced. The expression “taking it easy” implies a state of relaxation, freedom from tension, and even spontaneity. These characteristics align more with someone who might enjoy excitement rather than someone actively avoiding it. A closer examination of other robust violations associated with Item E107 supports this notion: Participants who embrace the idea of “taking it easy” also express a preference for “having a lot of fun” (E57) and “loving large parties” (E7). Simultaneously, “taking it easy” is linked with introversion through low activity levels (Facet 4) and low assertiveness (Facet 3). Importantly, these two facets reveal no monotonicity violations with Item E107 (see Fig 2).

It becomes evident that Item E107 is not adept at distinguishing extraverts from introverts, as also reflected in its low correlations with other extraversion items, ranging from -.13 to .22, with an average of .06. The factor loading of E107 on its respective facet is merely .24, suggesting potential issues from a factor analysis standpoint. Nevertheless, removing E107 from the factor model has minimal impact on the overall structure, with the facet of activity level changing only marginally from .44 to .45. While factor analysis may leave room for argumentation based on cutoff criteria or theoretical considerations, the results of state-trace analysis strongly suggest that Item E107 may not be justifiably retained in the questionnaire.

### 5.3 Discussion

The illustrative state-trace analysis of the IPIP-NEO-120 introduces a new perspective on navigating dimensional problems in psychological tests. Unlike factor analysis, which allows for interpretive flexibility, state-trace analysis mandates precise modifications. Sometimes these modifications have a significant impact on the factor analytic structure, as in the case of modesty-trust. Here, the factor loading of modesty underwent a remarkable transformation from .14 to .73. This improvement was not a product of trial and error or adherence to factor analytic criteria. State-trace analysis pinpointed the specific inconsistency: two modesty items were at odds with the trust facet. A semantic interpretation further revealed that these modesty items were indicative of another dimension: low self-confidence.

While alternative approaches might have uncovered and addressed the disparities between modesty and trust, the clarity and justification for resolution are notably more challenging to discern through the lens of factor analysis alone. This difficulty in identification and justification may shed light on why the original authors [3, 17] did not arrive at the seemingly straightforward solution.

In certain cases, factor analysis and state-trace analysis can lead to completely different conclusions. For example, Item C110 would never have been flagged as problematic by factor analysis due to its high factor loading. While the problem with C110 may not seem highly critical in practical terms, dismissing it altogether is not a viable option. Fixing Item C110 promises not only to increase its factor loading, but also to contribute to the theoretical understanding of the achievement striving facet.

State-trace analysis occasionally uncovers logical contradictions that are somewhat hidden, but expose significant theoretical challenges. Within the framework of the Big Five model, there is an implicit assumption that conservative individuals, on average, cannot be intellectual. This assumption, though hidden by factor analysis, becomes explicit when examined using state-trace analysis. While the majority of psychologists are likely to consider the Big Five model valid, it remains doubtful that many would assert the categorical notion that conservative individuals cannot possess intellectual qualities.

Finally, state-trace analysis emerges as a valuable tool when factor analysis yields items with low factor loadings, yet there are other compelling reasons to retain these items in the questionnaire. If these items fail to coalesce into a cohesive dimension with the others, it provides a strong argument for their removal.

Certain personality psychologists may raise objections, contending that the numerous violations observed in the IPIP-NEO-120 stem from its status as a still-developing questionnaire. Their argument might posit that the five-factor model with 30 facets holds value, but only the NEO-PI-R can accurately assess it. In the following section, I endeavor to demonstrate that, although empirically more challenging to support, the NEO-PI-R also contains items that violate unidimensionality within factors.

## 6 Part II: NEO-PI-R

The NEO-PI-R stands out as one of the most widely recognized questionnaires in the field of psychology, making a detailed introduction unnecessary (for an overview, see [4]). However, given its proprietary nature, there is a scarcity of freely available data from researchers. Consequently, the pool of available datasets for analysis is limited, often comprising only small samples (with *n* < 1, 000), which, in turn, requires to make certain methodological compromises.

### 6.1 Method

The NEO-PI-R comprises 240 items, with 48 dedicated to each dimension (eight for each of the six facets). This configuration results in a substantial number of pairwise tests, totaling 11,250. A dataset featuring raw item ratings from the Eugene Springfield sample is available with *N* = 857 [23]. Approximately 2% of the sample had more than 5% missing values, prompting their removal before state-trace analysis (*N* = 841).

Additionally, another dataset with *N* = 500 is accessible from a Netherlands-based study employing the Dutch version of the NEO-PI-R [21]. While a translation cannot be identical to the original test, the presence of problematic items in both versions serves as evidence of general semantic inconsistencies. The inherent limitations of small sample sizes, coupled with the necessity for *α* adjustment due to the multitude of tests, result in an inadequately small statistical power.

A more effective strategy is to assess each item against the entire factor, thereby reducing the number of tests to 240 and resulting in a more manageable *α* of .000208. However, this value is still relatively low for a dataset with *N* < 1, 000. To provide a more intuitive comparison: the power for a one-sided test with a correlation of .1, an *α* of .000208, and a dataset of *N* = 857 is merely 27%. But it is crucial to recognize that with 240 items being tested, there are numerous opportunities to encounter a significant result. On an aggregated level, it becomes increasingly likely that some of the more pronounced monotonicity violations will still be detected. Nevertheless, this approach represents a compromise, as many violations may go undetected. Another drawback is the challenge of pinpointing the precise source of the problem: whether it is an item violating monotonicity for a specific facet, specific items, or the entire factor itself.

An alternative approach is to draw on logical reasoning and prior findings. Since the IPIP-NEO-120 was designed as a substitute for the NEO-PI-R, it is reasonable to assume they share similar issues. A promising test candidate is the relationship between the facet liberalism (referred to as values in the NEO-PI-R) and intellect (termed ideas in the NEO-PI-R). In the IPIP-NEO-120 analysis, these two facets demonstrated a U-shaped relationship, which should be reproducible in the NEO-PI-R. To examine this, all items from the values facet can be collectively tested against all items from the ideas facet in a single analysis.

It is also viable to identify promising test candidates at the item level. Only two items meet the criteria of (a) violating monotonicity in the IPIP-NEO-120 analysis and (b) having nearly identical equivalents in the NEO-PI-R. These items are N51 and A84. Specifically, Item N51, “I rarely overindulge” closely resembles the N21 NEO-PI-R item. Similarly, Item A84, “I have a high opinion of myself” bears a striking resemblance to Item A144 in the NEO-PI-R. Verification of these correspondences require the reader to consult the copyrighted NEO-PI-R manual.

In the IPIP-NEO-120, N51 exhibited violations only in conjunction with the self-consciousness facet. Consequently, N21 of the NEO-PI-R can be directly tested against this facet to ascertain its compatibility. On the other hand, Item A84 in the IPIP-NEO-120 displayed multiple violations spanning different facets. Given its complete incompatibility with the trust facet, it is logical to test A144 of the NEO-PI-R against the trust facet. The previous analysis also sheds light on why A144 should be considered problematic: it not only measures modesty but also reflects low self-confidence.

Before delving into these more targeted analyses, it is crucial to acknowledge that the NEO-PI-R datasets have a sample size approximately 700 times smaller than that of the IPIP-NEO-120. Even if both questionnaires share similar issues, detecting them in the NEO-PI-R datasets might prove challenging due to the considerably smaller sample size. To gauge the potential power constraints, a bootstrapped power analysis was conducted using the IPIP-NEO-120 dataset. Specifically, 857 participants were randomly selected, and from this sample, 1,000 bootstrap samples were drawn. Significance was tested at *α* = .017 (correcting for three tests: facet liberalism, Item N51, and Item A84). This entire procedure was repeated 1,000 times, resulting in a proportion of significant results. The power was 68% for the facet liberalism, 14% for Item N51, and 79% for Item A84. Consequently, it may not be meaningful to test Item N51 in a sample of 857 or fewer participants. As a result, only the equivalents of the facet liberalism and Item A84 will be tested in the NEO-PI-R.

An additional challenge associated with small datasets is the potential scarcity of values for certain scale points. In the two datasets under examination, approximately 50 items recorded fewer than five responses on at least one of their scale points. To mitigate the risk of obtaining spurious results, these scale points were excluded before the analysis (refer to the General Method section). Consequently, it remains uncertain whether these items genuinely exhibit monotonic relationships with others. A comprehensive resolution to this quandary would entail working with larger samples or adopting an alternative methodology, and these aspects are deliberated upon in the General Discussion.

In summary, each dataset undergoes two specific tests: (1) facet values versus facet ideas and (2) Item A144 versus facet trust. Considering there are two tests, *α* is adjusted to 0.025. Furthermore, each of the 240 items will be individually tested against the corresponding factor in a separate analysis, with an *α* of 0.000208.

### 6.2 Results

To begin, the findings for Item A144 (akin to A84 in the IPIP-NEO-120, “I have a high opinion of myself”) are presented. The significance test for the Eugene dataset yields a residual deviance of 92.051 and a *p* value of <0.0001. In the Dutch dataset, the deviance is 22.222, and the *p* value is .019. Both tests demonstrate statistical significance, reinforcing the earlier conclusion that “thinking highly of oneself” is not solely measuring modesty.

**Fig 8:**
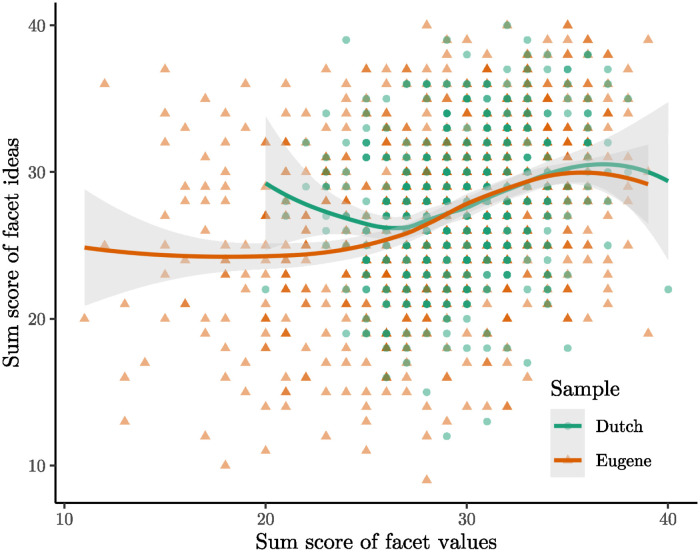
Facet ideas plotted against facet values. LOESS smoothing was applied. Shaded regions show 95% confidence intervals.

Moving on, the examination of the facet values versus the facet ideas is presented. In the Eugene sample, the statistically non-significant deviance of 75.136 with a *p* value of 0.5395 is observed. Conversely, for the Dutch sample, the deviance is 199.43, with a statistically significant *p* value of <0.00001. Fig 8 illustrates the relationship between both facets. Due to the small sample sizes, numerous categories have scant values, rendering it impractical to plot means and confidence intervals. Instead, a scatter plot with LOESS smoothing was generated. In the Eugene sample, the relationship appears monotonic, but the conservative segment of the scale is flat. For the Dutch sample, the relationship is slightly U-shaped, indicating a violation of monotonicity. Notably, the very conservative part of the scale is entirely absent, yet the violation remains pronounced. The sum scores for the facet values from 20 to 26, on average, yield excessively high sum scores on the facet ideas. This suggests that somewhat conservative individuals exhibit higher intellectual traits than the Big Five model permits.

Given that both datasets consist of student samples, the underrepresentation of conservative individuals is not unexpected. Before arriving at any conclusive interpretations, it is imperative to investigate a more diverse sample. However, considering the lower power for the NEO-PI-R data sets and the compelling evidence from the initial analysis on the IPIP-NEO-120, it seems probable that a non-monotonic relationship between values and ideas will be identified again in the future.

Following the presentation of the specific hypotheses, the individual item tests are showcased. Among all item tests against the corresponding factor, nine are significant for the Eugene dataset (Table 1), and 19 for the Dutch dataset (Table 2). The intersection between the Eugene and Dutch samples includes four items: A164, E17, C35, and N96. These items are particularly noteworthy as they markedly violate monotonicity across two samples and two languages.

**Table 1:** Items that significantly violate monotonicity in the revised NEO personality inventory (Eugene springfield sample) at an *α* of .000208.

Item	*p*	Deviance
A129	< 0.00005	144.023
A144	< 0.00005	133.056
A164	< 0.00005	71.151
A99	< 0.00005	90.728
C35	< 0.00005	119.120
E17	< 0.00005	227.410
E2	< 0.00005	150.260
N31	0.00015	98.040
N96	< 0.00005	179.024

*Note.* Item designations A, C, E, N, and O stand for the factors agreeableness, conscientiousness, extraversion, neuroticism, and openness, respectively.

**Table 2:** Items that significantly violate monotonicity in the revised Dutch NEO personality inventory data set at an *α* of .000208.

Item	*p*	Deviance
A164	0.00010	85.017
C35	< 0.00005	267.280
E17	< 0.00005	558.276
E47	0.00005	111.230
E167	0.00020	130.095
N46	< 0.00005	181.052
N96	0.00015	121.697
N111	0.00005	119.596
N141	< 0.00005	214.922
O18	< 0.00005	111.985
O88	< 0.00005	151.246
O103	< 0.00005	206.220
O113	< 0.00005	129.932
O133	< 0.00005	90.120
O163	< 0.00005	91.323
O168	0.00010	121.744
O173	< 0.00005	126.816
O193	0.00015	116.515
O208	< 0.00005	157.960

*Note.* Item designations A, C, E, N, and O stand for the factors agreeableness, conscientiousness, extraversion, neuroticism, and openness, respectively.

**Fig 9:**
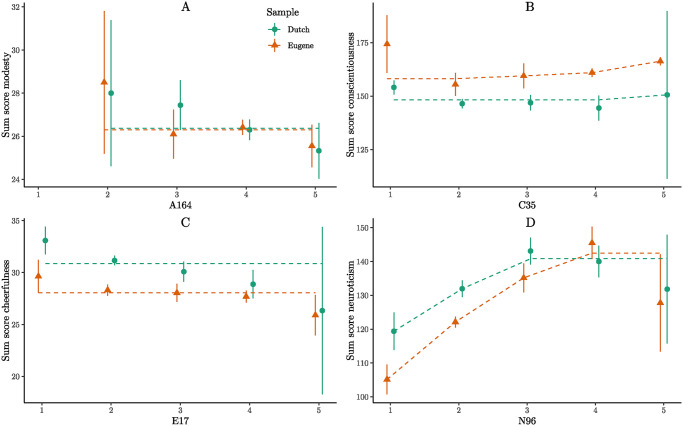
Exemplary violations of monotonicity in the NEO-PI-R. Displayed are mean values with 95% confidence intervals for two samples of the NEO-PI-R (Eugene and Dutch). Note that for subfigure A, only 1 person chose answer option 1 for A164, so the data for this category is not displayed. The dashed lines represent the best fitting isotonic models as a visual guide.

Item A164 pertains to altruism, yet its wording carries connotations of arrogance. A rephrased version could be: “Many people like me.” While this statement may capture altruistic tendencies, it also functions as an effective reverse-coded modesty item. Evidently, the relationship to the sum score of modesty is negative (Fig 9A). A164 encapsulates two conflicting facets of agreeableness: the inclination towards altruism while lacking modesty. Therefore, the item necessitates revision to convey altruism without sounding arrogant.

Item C35 addresses civic duties such as voting. Some participants opted for an extreme response, indicating a lack of seriousness towards these duties (scale point 1). Strikingly, these participants scored, on average, higher on conscientiousness compared to those choosing other C35 scale points, as depicted in Fig 9B. Consequently, these individuals exhibited conscientious traits, yet harbored a dislike for civic duties, or more specifically, voting. The act of voting might not be a reliable indicator of conscientiousness. It is also noteworthy that C35 is categorized under the facet of competence, despite voting not necessitating expertise.

Item E17 bears a resemblance to Item E107 of the IPIP-NEO-120 (“I like to take it easy”), which emerged as one of the most problematic items in the first analysis. A person who is relaxed can exhibit traits of both extraversion (spontaneity, having fun) and introversion (low activity level, low assertiveness), contingent on the context. This same ambiguity appears to afflict Item E17 of the NEO-PI-R. For instance, Fig 9C illustrates that this item is negatively associated with the facet cheerfulness.

Lastly, N96 evaluates angry hostility, gauging a person’s temperamental disposition. As depicted in Fig 9D, highly temperamental individuals exhibit lower levels of neuroticism than expected. This problem is intriguing as it proves challenging to identify through factor analysis. The correlation between Item N96 and the neuroticism sum score is .45 for the Eugene sample and .3 for the Dutch sample. Although the item may function adequately in factor analysis, it still appears unsuitable for inclusion in the neuroticism scale. Please note that a more detailed discussion of the item is difficult due to copyright restrictions of the NEO-PI-R, which prevent a direct and comprehensive elaboration in this context.

### 6.3 Discussion

At first glance, the NEO-PI-R may appear to be more robust than the IPIP-NEO-120 due to fewer observed violations of monotonicity. However, this conclusion is misleading. The limited size of the data sets and the less sensitive tests make it unlikely to detect small and medium violations of monotonicity, potentially leading to numerous misses. Only violations of substantial magnitude can be detected statistically. However, given the widespread use of the NEO-PI-R, even a few clearly identified problematic items can have a significant impact.

The NEO-PI-R is one of the most successful questionnaires in psychology, with the seminal publication [4] accumulating approximately 38,000 citations according to Google Scholar. It has been translated into 40 languages and used beyond research settings, and has likely been administered hundreds of thousands, if not millions, of times. Despite its widespread use, the questionnaire contains items that are inconsistent with a five-factor model. This observation does not impugn the overall quality or utility of the questionnaire; if the remaining items demonstrate validity, the impact of errors introduced by problematic items may be limited. Nevertheless, these errors are avoidable, and the items in question represent significant logical inconsistencies. Such inconsistencies are not readily apparent from factor analysis, or they would likely have been corrected. Thus, state-trace analysis brings a new perspective to even well-established questionnaires in psychology. The practical implications of this conclusion become clear when examining an updated version of the NEO-PI-R, namely the NEO-PI-3 [24].

The NEO-PI-3 stands as a more accessible alternative to the NEO-PI-R, designed for adolescents [24]. However, the authors also considered it as a potential replacement for the NEO-PI-R in the future. While the NEO-PI-3 is primarily based on the NEO-PI-R, 37 items underwent rephrasing or replacement. Notably, two problematic items discussed earlier, C35 and E17, were modified in the NEO-PI-3. While this suggests instances where factor analysis and state-trace analysis align, a more nuanced perspective emerges upon scrutinizing the specific alterations made in the NEO-PI-3.

For example, instead of examining the problems with item C35, [24] chose to replace it with an entirely different item. There was no exploration of the paradox of why people who “do not take voting seriously” are highly conscientious. The decision to replace C35 appears to have been motivated by its item-total correlations falling below .3: .29 for Form S and .25 for Form R [24] (p. 265). In the NEO-PI-3, however, these values did not change substantially: .28 for form S and .4 for form R. The original version of C35 remains part of the regular NEO-PI-R, and from a factor-analytic perspective, there is no clear rationale for its removal. In contrast, state-trace analysis indicates that the item cannot be part of the conscientiousness dimension.

A similar argument applies to item E17, which underwent only minor rephrasing in NEO-PI-3 and, given its semantics, probably still violates monotonicity. Item-total correlations improved slightly but remained unsatisfactory: from .07 to .15 for Form S and from 0 to .06 for Form R [24] (p. 265). The original authors seem to be unaware that “being relaxed” is semantically ambiguous, representing a potential sign of either extraversion or introversion, depending on the context.

While there may be instances where factor analysis and state-trace analysis agree, the conclusions drawn seem to differ significantly. Moreover, obvious problems with items A144, A164, and N96 were not even identified by [24].

The NEO-PI-R is likely to contain numerous additional nonmonotonic items, but their identification requires a more robust methodology. This can be accomplished by (a) increasing the sample size, (b) formulating more precise item tests rooted in semantic theorizing, or (c) adopting an experimental approach to establish a balanced design and increase internal validity. These considerations are the focus of the general discussion.

## 7 General discussion

In this paper an alternative method for questionnaire validation has been proposed—state-trace analysis. This distinctive approach provides a means of testing whether a set of items can be effectively reduced to a single factor. The capabilities of state-trace analysis were illustrated by analyzing two five-factor model questionnaires based on 30 facets in three samples. The primary goal was to introduce the method of state-trace analysis to personality research by showing different ways of conducting the analysis, presenting the results, and interpreting the findings.

Overall, the results are promising. State-trace analysis successfully identified numerous problematic items that questionnaire developers have overlooked using traditional methods. Specifically, the IPIP-NEO-120 revealed several weaknesses that require attention and rectification. While the NEO-PI-R appears to have fewer issues, the smaller sample sizes in the datasets raise concerns about potential false negatives (missed problems). These methodological challenges, along with potential solutions, are discussed in detail. Before delving into the discussion, a summary highlighting the benefits and limitations of state-trace analysis based on the obtained results is presented.

### 7.1 Benefits of state-trace analysis

What does state-trace analysis offer over existing psychometric methods? First and foremost, it provides a general mathematical solution to unidimensionality with minimal assumptions. There is no need for unwarranted adherence to linear or logistic relationships between items and latent variables; any kind of monotonic relationship will suffice. In the future, psychologists may find state-trace analysis to be the most general and comprehensive approach, making other tests of unidimensionality unnecessary. It is important to note, however, that this does not imply the complete obsolescence of other methods, nor does it imply that other methods are always in conflict with state-trace analysis.

For example, when correlations or factor loadings are exceptionally high, monotonicity is typically not violated, making factor analysis a suitable choice. However, such high correlations are rare in psychology. The examination of Item C110 illustrates that a factor loading of .65 can be attained even when monotonicity is clearly violated. In essence, state-trace analysis proves to be a more sensitive test of unidimensionality than factor analysis.

The lower the correlations, the more sensitive state-trace analysis becomes compared to factor analysis. For example, if the correlation between two items is only.3, the relationship may or may not be monotonic. Relying on a correlation alone provides little insight into unidimensionality and increases the potential for erroneous conclusions. The state-trace analysis of the IPIP-NEO-120 indicates that 28 items need to be modified or removed. In contrast, the factor structure presented by [17] contains only two items (E107, A114) with item factor loadings below .3, a common cutoff criterion (see, for example, [25]). However, even these two items were not altered or removed from the questionnaire by the test authors.

There may be valid reasons for retaining items with low factor loadings, such as limited alternatives or theoretical justifications. Factor-analytically, there is no inherent problem with this practice, provided the sample size is large enough to ensure reliability. However, from a unidimensionality perspective, the question remains whether retaining a low loading item is a mistake. In particular, state-trace analysis makes it clear that item E107 should be removed, while item A114 should be retained. A conclusion that would be difficult to defend with factor loadings alone.

It is difficult to imagine a scenario in which factor analysis (or any other method) would outperform state-trace analysis in assessing unidimensionality. However, a synergistic approach is conceivable in which both methods complement each other. Factor analysis can help reveal general semantic structures, while state-trace analysis serves as a rigorous test of unidimensionality.

### 7.2 Limitations of state-trace analysis

State-trace analysis is likely the most general conceptualization of unidimensionality and appears to have no major drawbacks, particularly when compared directly to alternative methods.

Although this article has emphasized multiple times that large sample sizes are necessary to draw robust conclusions, this is not an inherent limitation of state-trace analysis itself but rather a consequence of the numerous tests involved when analyzing item pairs. In this paper, *α* correction was rigorously applied, leading to exceptionally low thresholds of 0.0000181 (for the IPIP-NEO-120) and 0.00208 (for the NEO-PI-R), which require large sample sizes for adequate power. However, if the standard 0.05 threshold is used, this issue does not arise. The author does not advocate for indiscriminately increasing the *α* level; instead, the recommendation is to either aim for large sample sizes—irrespective of the specific method employed (e.g., factor analysis, IRT, or state-trace analysis)—or to concentrate on testing a small set of highly specific hypotheses.

A practical challenge in state-trace analysis arises when certain scale points receive very few responses. This can lead to the emergence of extreme monotonic models that may not reflect realistic patterns. A straightforward solution is to exclude these problematic scale points from the analysis. Alternatively, a balanced experimental design can be employed to address this issue (see also the Section *Experimental and Balanced Approaches*). It is worth noting that this problem is not unique to state-trace analysis and also affects other methods. For example, correlation coefficients can be significantly distorted by outliers, and removing these outliers can lead to substantial changes in the computed coefficient, in turn affecting results of factor analysis.

One theoretical drawback is that if the relationship between the latent and manifest variables is more specific than monotonic, the method’s effectiveness could be diminished by assuming monotonic functions. For instance, if there is reason to believe the relationship is linear, it would be more appropriate to test for linearity between the manifest variables rather than relying solely on monotonicity. However, this is not truly a limitation, as state-trace analysis is a highly general method, and its algorithms can be adapted to accommodate specific relationships (for an example see [11] p. 43). Furthermore, when analyzing the data, a linear relationship between manifest variables would typically become evident, allowing adjustments to be made accordingly.

When evaluating state-trace analysis without directly comparing it to alternatives, some general logical limitations become apparent. For example, the principle of falsification applies: discovering a non-monotonic relationship between two manifest variables allows the conclusion that unidimensionality is violated. However, finding a monotonic relationship does not confirm unidimensionality; it merely fails to provide evidence to the contrary. This lack of contradiction remains an important argument, especially when the result is consistently replicated. Nevertheless, accumulating evidence does not eliminate the possibility that two dimensions could appear indistinguishable from a single dimension under specific conditions.

To illustrate, consider again the analogy of travel distance and time: if speed is held constant, the two-dimensional relationship between distance and time is obscured, leading the researcher to mistakenly infer a unidimensional structure. Consequently, conclusions must be drawn with caution, particularly given that the data analyzed here was not obtained experimentally, precluding any establishment of causality (see also the Section *Experimental and Balanced Approaches*).

This limitation intersects with the broader debate about whether state-trace analysis can identify the number of underlying *systems* or *processes* involved. [13] argue that these terms are poorly defined, rendering the method unsuitable for addressing such vague questions. Nonetheless, they note that “STA might be used to conclude that the data are not complex enough to rule out a single varying parameter” (p. 16), advocating for the use of the more neutral term *parameter*. In psychological questionnaires, there is little need to invoke ambiguous concepts like *systems* or *processes*; instead, the focus can remain on a single parameter representing the latent variable, simplifying interpretation and maintaining conceptual clarity.

Finally, the assumption of monotonicity is itself subject to debate. [13] contend that this assumption unduly limits the applicability of state-trace analysis, particularly in the domain of cognitive psychology. However, compared to the restrictive assumptions often required by alternative methods, monotonicity is fairly modest. While it may not hold for certain complex models in cognitive psychology, it is easily justified in the context of psychological questionnaires.

In this context, it is crucial to recognize that if monotonicity between latent and manifest variables does not hold, a monotonic relationship between manifest variables cannot reliably serve as evidence for a single dimension [13]. Nonetheless, this concern is unlikely to affect the typical use case of questionnaire items, where monotonicity is generally a reasonable and defensible assumption.

### 7.3 Approaches for large and small data sets

Overall, more problems were identified in the IPIP-NEO-120 than in the NEO-PI-R. However, comparing the two questionnaires based on samples of different sizes is not entirely fair. There are distinct differences between analyzing the monotonicity of a large dataset and that of a small one.

The analysis of the IPIP-NEO-120 was specifically designed for large datasets. The significance level was adjusted without compromising statistical power, and every pair of items was tested. A network graph was utilized to visualize all violations, allowing the identification of the most problematic items. This approach represents the ideal scenario, and for nonproprietary questionnaires, it is relatively straightforward to collect large samples online. For example, [26] provided data on 15 psychological questionnaires, each with a sample size exceeding 200,000 participants. Similar methodologies can be applied to assess the unidimensionality of these questionnaires, following the approach used for the IPIP-NEO-120.

It should be acknowledged that due to the sheer size of the sample, the IPIP-NEO-120 is susceptible. The null hypothesis posits that items are monotonically related. With large samples, even minute monotonicity violations can achieve statistical significance. While this sensitivity is beneficial for scientific rigor, it may conflict with the practical priorities of questionnaire developers. Creating a scale that is unequivocally unidimensional becomes more challenging with large samples—though not impossible. For instance, in the IPIP-NEO-120 analysis, the dimensions of conscientiousness and neuroticism achieve perfect unidimensionality if just two items are removed. Yet it is far more convenient to utilize small samples, which reduces power and thereby prevents rejecting the null hypothesis. In this context, proprietary questionnaires enjoy an inherent advantage, as the cost of acquiring large samples is often prohibitively high—except, perhaps, for the test authors themselves.

Hence, it is unsurprising that the smaller NEO-PI-R datasets resulted in fewer instances of unidimensionality violations within factors. In small samples, power significantly diminishes, making it impractical to test all possible item pairs. This challenge intensifies with the length of the questionnaire, placing the NEO-PI-R at an advantage regarding the null hypothesis, given its double length compared to the IPIP-NEO-120. Enhancing power can be achieved by testing an item against the entire factor simultaneously, mitigating the need for an excessively stringent *α* adjustment. However, a more effective alternative to boost power involves conducting more specific tests, necessitating substantial theoretical groundwork, including semantic analyses of items.

### 7.4 Semantic analyses

Several post-hoc (inductive) semantic analyses of specific item pairs have already been presented in this article. For instance, the unexpected positive association between “thinking highly of oneself” and the facet *trust* challenges the five-factor model, which predicts a negative relationship. Semantically, however, this result is not entirely surprising. According to [27], individuals with high self-esteem are more likely to engage in self-revealing behavior in relationships, driven by their trust in their partners. Additionally, in group contexts, trustworthy individuals are often perceived as having high self-esteem [28]. In essence, trusting others requires a certain degree of self-esteem. A thorough semantic analysis could potentially have anticipated this discrepancy.

A simple heuristic is to always consider whether scenarios can be identified that violate monotonicity. For instance, “being relaxed” can be associated with high or low extraversion, depending on the context (relaxed in the sense of being loose and natural or in the sense of being calm). The five-factor model assumes that “being relaxed” can only be a sign of low extraversion (reduced activity).

A systematic approach to identifying such problems a priori (deductively) is the DELPHI method [29], which involves a group of experts working toward a consensus on items for a questionnaire. While the method primarily emphasizes content validity, additional criteria, such as monotonicity, can also be considered. By reflecting on whether all items could be monotonically related to the latent variable and to one another, scale developers could identify potential violations of unidimensionality early in the scale development process.

The method of discriminant content validity [30] adopts a similar approach, involving experts in evaluating content validity. However, it goes a step further by also assessing how well items reflect competing constructs, aiming to identify potential multidimensionality. This method could be enhanced by incorporating insights from state-trace analysis and explicitly integrating the monotonicity criterion. Experts could be instructed to consider scenarios where item pairs might exhibit non-monotonic relationships. If such scenarios can be conceived, it would indicate that the items are not unidimensional.

With a coordinated effort, it should be possible to identify more semantic inconsistencies before items are empirically tested. In addition, if some items remain ambiguous, specific hypotheses about them can be tested with much smaller samples than in an inductive approach where all items must be tested. It is important, however, not to view large-sample inductive approaches and small-sample deductive approaches as mutually exclusive. In this paper, the inductive analysis of the IPIP-NEO-120 laid the foundation for testing specific hypotheses in the NEO-PI-R, and this approach proved successful: 3 out of 4 tests were statistically significant, consistent with the expected average power. Further improvements to these specific hypothesis tests can be achieved by incorporating experimental designs.

### 7.5 Experimental and balanced approaches

An experimental state-trace analysis can be executed in the same manner as any other experiment in personality psychology—using vignettes [31]. For instance, to explore the relationship between the facets of modesty and trust, vignettes describing individuals varying in levels of agreeableness (ranging from generally agreeable to not agreeable, with variations in between) can be employed. Subsequently, modesty and trust items can be assessed as dependent variables, and standard state-trace analysis can be applied to this experimental data. The only distinction from the analyses presented here lies in calculating mean values for the dependent variables by grouping them based on the independent variable. Otherwise, the methodology and inference statistics remain the same.

If the anticipated relationship between two dependent variables is not expected to be clearly negative, the experimental setup becomes more intricate. Two independent variables are required that differentially influence the latent variables [11]. However, this is essentially a matter of identifying suitable vignettes, which, in turn, relies on robust theoretical groundwork. The semantic analyses presented in this paper can serve as an initial reference. For example, one could experimentally examine the hypothesized U-shaped relationship between liberalism and intellect. A potentially insightful vignette might involve describing a priest who, by definition, is conservative (e.g., believes in the authority of religion) but is also intellectual (engages in theology and philosophy).

The experimental design offers two key advantages: (1) it enables causal inference, and (2) each participant can be individually tested. This approach allows for the identification of specific participants for whom a unidimensional model does not hold (on pp. 73–82 [11] provide an illustrative state-trace analysis on the individual level). Subsequently, these participants can be interviewed to gain a deeper understanding of the semantic inconsistencies.

Another advantage of the experimental design is the consistent sample size across all conditions, preventing cases where there are insufficient participants responding at the extreme scale points.

A notable disadvantage is the challenge of accurately assessing a person’s personality based on a brief description. Experiments with vignettes may not always yield the same results as those obtained in nonexperimental studies. It is reasonable to argue that each method carries distinct strengths in terms of validity, with external (representative of real life) and internal (causality) validity often appearing mutually exclusive. Nevertheless, when both nonexperimental and experimental studies converge on the same conclusion, a robust foundation can be established.

If external validity is deemed more crucial than internal validity, maintaining a balanced design remains desirable. This can be accomplished through an unconventional methodology in which participants receive instructions similar to:

Envision the individual closest to you, someone you know intimately (e.g., your partner or a family member). For each of the following statements, assess whether you or your close associate would be more inclined to agree.

Given that the participant and the close person diverge on the latent variable, items associated with this latent variable should consistently maintain the same order. For example, if the participant exhibits higher extraversion than the close person, the participant should consistently assign higher ratings to themselves on all extraversion items. The data analysis becomes a matter of identifying items that consistently deviate from the expected order across participants.

This approach is advantageous as it is inherently balanced, with a 50% chance, on average, of being below or above the close person on the latent variable (for a reasonably representative sample). Moreover, the methodology is straightforward, involving only three options for each item (for persons *A*, *B* : *A* > *B*, *B* > *A*, or *A* = *B*. One potential disadvantage is that if people are very close on the latent variable, no meaningful information can be derived.

A specific example could involve testing the facet of liberalism against the facet of intellect. According to the five-factor model, an increase in liberalism should correspond to an increase in intellect. If person *A* consistently asserts that she is more liberal than person *B*, but less intellectual, this presents a logical contradiction. If such contradictions are observed for multiple participants, it challenges the validity of the proposed unidimensional model. This novel design could prove to be an efficient way to generate items since logical inconsistencies can be detected early in the research process with only a few participants. However, it is important to note that the described method is primarily effective in identifying problematic items and may not be suitable for scaling participants.

In summary, each approach comes with its own set of strengths and weaknesses, making them complementary rather than exclusive. The inductive, nonexperimental approach presented in this paper serves as a solid foundation for delving deeper into the issues of the IPIP-NEO-120 and NEO-PI-R through deductive experimental and balanced designs. The extension of these methods to other psychological questionnaires and tests is a natural progression.

### 7.6 Representativeness

Every statistical test relies on the assumption that the sample is drawn in a representative manner from a population. However, the specific population from which the samples studied here were drawn is not clearly defined. While this issue is more methodological than related to dimensionality, it warrants attention. For instance, the IPIP dataset was collected from the internet, resulting in a highly diverse sample. On the other hand, the NEO-PI-R datasets were obtained from U.S. and Dutch student samples. It is challenging to assert the superiority of one sample over the other, and none of them can be deemed ideal in terms of representing the general population. Nevertheless, if the criterion is the demonstration of the validity of the five-factor model, they can be considered reasonably good.

In all three samples, establishing a five-factor structure is straightforward [3, 21, 32]. The five-factor model appears valid from a factor analytic perspective. However, state-trace analysis contradicts this established five-factor structure. The lack of representativeness of the sample for the general population applies to both factor analysis and state-trace analysis. When the two analyses contradict each other, the blame cannot be placed on the representativeness of the sample.

### 7.7 Alternatives to state-trace analysis

Several methods have been proposed for assessing unidimensionality in the past [7, 8, 33–35], yet none quite attains the generality and simplicity embodied in state-trace analysis. State-trace analysis distinguishes itself with its minimal, plausible, and explicit assumptions. In contrast, alternative methods face logical challenges by presuming very specific relationships between variables. There is no compelling reason to assume, for instance, that latent extraversion is *linearly* tied to manifest extraversion items. This inherent issue casts doubt on the very popular factor-analytical methods. Notably, many psychologists have expressed dissatisfaction with existing approaches for analyzing unidimensionality [7, 8, 33, 34].

If state-trace analysis represents the most comprehensive mathematical depiction of unidimensionality, true alternatives would inherently be specific instances within this general framework. To illustrate, consider Guttman scaling [36, 37], which stands out as one of the rare approaches addressing unidimensionality without relying on factor analysis.

If Guttman scaling indicates a perfectly unidimensional scale, the latent variable and the manifest variables exhibit a monotonic relationship [38]. Consequently, state-trace analysis will yield a unidimensional model as well. Guttman scaling, in essence, can be viewed as a special case within the broader framework of state-trace analysis. However, it is somewhat less appealing as a special case due to certain methodological limitations it carries.

In its original form, Guttman scaling is exclusively applicable to dichotomous items [38]. Extensions to polytomous items have been introduced subsequently [37, 38]. However, the associated significance tests introduce an unfavorable null hypothesis: In Guttman scaling, the data is tested against independence (items are completely unrelated). Consequently, the test will likely yield significance against null even when there is only some degree of monotonicity in the data. This characteristic renders it challenging to identify problematic items, such as C110 (refer to Fig 5), which exhibits substantial monotonic trends without being entirely monotonic.

The significance tests used in Guttman scaling seem to be a pragmatic compromise in the absence of a definitive solution. As noted by [38] (p. 44), about 30 model tests have been proposed in the 50 years since the method was introduced. Guttman scaling lacks the conceptual idea of the state-trace, making it difficult to identify an appropriate null hypothesis.

Another limitation of Guttman scaling is that it is restricted to discrete data. It cannot be used with continuous variables, such as reaction times, which are common in psychology, for example, in implicit association tests and time-based cognitive assessments. Overall, as a special case of state-trace analysis, Guttman scaling does not seem to offer any distinct advantages.

This is similar to other special cases of state-trace analysis, such as factor analysis and item response theory, both of which impose very specific monotonic relationships between latent and manifest variables. However, this comes at a significant cost, as the assumption of such strict relationships is likely to be incorrect and cannot be empirically tested because of the problem of nomic measurement (see Section *Monotonicity: One Criterion to Rule Them All*). Nevertheless, if there is some indirect justification for a particular relationship—whether linear (factor analysis), logistic (item response theory), or step-wise (Guttman scaling)—these methods would be more sensitive in capturing violations of unidimensionality. If there is no such justification—which is almost always the case—these alternatives will remain the second- or third-best choice to assess unidimensionality.

### 7.8 Conclusion

State-trace analysis offers a fresh perspective on psychological measurement by providing a robust framework for rigorously examining unidimensionality. Despite its minimal and straightforward assumptions, the method yields powerful insights, making it easier to identify items within a factor that deviate from unidimensionality.

This paper has presented illustrative analyses of the five-factor model, with results highlighting the need for modifications to numerous items and facets to preserve the integrity of the five dimensions. These findings challenge longstanding conclusions drawn from traditional methods, potentially signaling a shift in personality research—and psychology more broadly—away from the dominance of factor analysis.

Continued methodological advancements in state-trace analysis promise to equip psychologists with more refined and effective tools for constructing robust questionnaires, paving the way for greater precision in psychological measurement.

## Supporting information

S1 Fig Structural equation model for the factor agreeableness including all modesty items. (PDF)

S2 Fig Structural equation models for the factor agreeableness excluding modesty items A54 and A84. (PDF)
