# Supplementary figures and images for: State-trace analysis meets personality measurement: Why the Big Five tests are not based on five latent dimensions and how to fix them
Source: PLoS One. 2025 Feb 19;20(2):e0317144. doi: 10.1371/journal.pone.0317144 (PMC11838909; doi:10.1371/journal.pone.0317144)

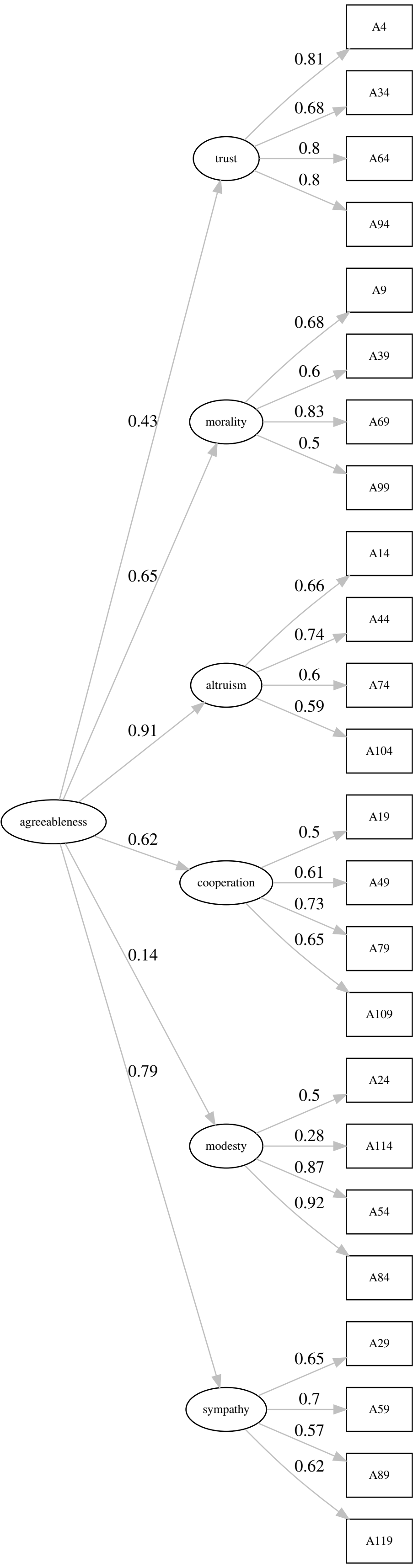

Supplement: S1 Fig — (PDF) [file pone.0317144.s001.pdf]

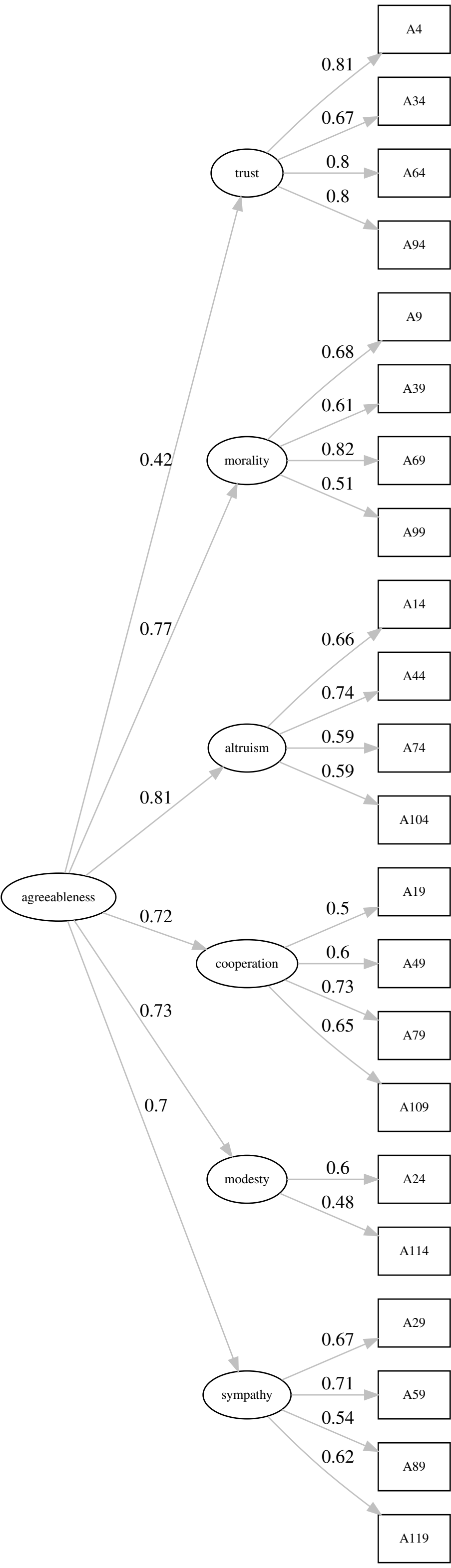

Supplement: S2 Fig — (PDF) [file pone.0317144.s002.pdf]
